# Long-Term Safety of Endoscopic Biliary Stents for Cholangitis Complicating Choledocholithiasis: A Multi-Center Study

**DOI:** 10.3390/jcm9092953

**Published:** 2020-09-12

**Authors:** Wisam Sbeit, Tawfik Khoury, Anas Kadah, Dan M. Livovsky, Adi Nubani, Amir Mari, Eran Goldin, Mahmud Mahamid

**Affiliations:** 1 Department of Gastroenterology, Galilee Medical Center, Nahariya 2221006, Israel; wisams@gmc.gov.il (W.S.); anas18kadah@msn.com (A.K.); 2 Faculty of Medicine in the Galilee, Bar-Ilan University, Safed 13100, Israel; amir.mari@hotmail.com; 3 Gastroenterology Department, Sha’arei Zedek Medical Center, Jerusalem 9103102, Israel; danlivo@szmc.org.il (D.M.L.); adinubani@gmail.com (A.N.); erang@szmc.org.il (E.G.); mahmudmahamid@yahoo.com (M.M.); 4 Faculty of Medicine, Hebrew University of Jerusalem, Jerusalem 9112102, Israel; 5 Gastroenterology and Endoscopy Units, The Nazareth Hospital, EMMS, Nazareth 16100, Israel

**Keywords:** cholangitis, safety, long-term, biliary, stents

## Abstract

Background: Treatment of cholangitis complicating choledocholithiasis includes biliary sphincterotomy and stone extraction. In certain cases of elderly comorbid patients with high risk for definitive endoscopic treatment, biliary stenting is the only measure for relieving biliary obstruction. Aim: We aimed to report the safety of retained biliary stone. Methods: a multi-center, retrospective case-control study conducted at two Israeli medical centers from January 2013 to December 2018 including all patients 18 years of age or older who underwent ERCP and biliary stent insertion for the treatment of acute cholangitis due to choledocholithiasis. Results: Three-hundred and eight patients were identified. Eighty-three patients had retained long-term biliary stents of more than 6 months (group A) from insertion compared to 225 patients whose biliary stents were removed within a 6-month period (group B). The mean follow-up in group A was 66.1± 16.3 vs. 11.1 ± 2.7 weeks in group B. Overall complications during the follow-up were similar between groups A and B (6% vs. 4.9%, OR 1.24, Chi square 0.69). Similarly, the rate of each complication alone was not different when comparing group A to group B (3.6%, 1.2% and 1.2% vs. 2.7%, 0.44% and 1.8%) for cholangitis, stent related pancreatitis and biliary colic, respectively (Chi square 0.85). Even after 12 months, the rates of overall complications and each complication alone were not higher compared to less than 12 months (Chi square 0.72 and 0.8, respectively). Conclusion: endoscopic biliary stenting for cholangitis complicating choledocholithiasis is safe for the long-term period without increase in stent related complications.

## 1. Introduction

Endoscopic retrograde cholangiopancreatography (ERCP) is considered an essential procedure for the treatment of numerous pancreatic and biliary tract conditions [1], mainly used for the management of choledocholithiasis complications [2,3]. Given its diagnostic and therapeutic potential coupled with its acceptable complications risk profile, ERCP has largely replaced surgical exploration of the common bile duct (CBD). ERCP has been reported to be successful in 80–95% of cases [4,5]. The therapeutic interventions used to treat choledocholithiasis include papillotomy, basket and or balloon extraction and in cases of large solid stones, lithotripsy is used.

In patients with septic cholangitis, most endoscopists prefer to perform endoscopic drainage via stenting without performing papillotomy [6]. Moreover, endoscopic insertion of biliary endoprosthesis has been proposed as an alternative treatment for elderly patients or those with high surgical risks [7,8]. Stent related complications are divided into early and late, the former including cholangitis, pancreatitis, perforation and bleeding and the latter including stent occlusion, migration, cholangitis and cholecystitis [9]. It is advocated that plastic stents should be removed or replaced within 3–6 months after insertion [10,11,12], while metallic stents are recommended to be replaced after 10 months to minimize the risk of stent occlusion and cholangitis [13]. Several studies have shown the beneficial effect of short-and long-term biliary stenting for irretrievable CBD stones in high-risk elderly patients [7,8,14]. However, only few studies have reported long term complications; stent migration and cholangitis were the main complications with prolonged stenting duration [15]. Previous studies have reported cholangitis to be the most common long-term complication of biliary stent in the elderly approaching 40% of patients, and 16% of patients died because of biliary-related causes [16]. Given the paucity of data regarding retained biliary stents beyond the recommended period of extraction or replacement, we aimed to report our experience regarding the long-term safety and complications of retained biliary stents in elderly patients hospitalized with cholangitis complicating choledocholithiasis and treated by biliary stenting.

## 2. Materials and Methods

We performed a multi-center, retrospective case-control study conducted at two Israeli medical centers (Galilee Medical Center and Sha’arei Zedek Medical Center from January 2013 to December 2018). Inclusion criteria for the study endpoints included: patients 18 years of age or older who underwent ERCP and biliary stent insertion for the treatment of acute cholangitis complicating choledocholithiasis. Exclusion criteria included patients with cholangitis secondary to biliary disease other than choledocholithiasis such as malignancy and biliary stricture, patients with Primary Sclerosing Cholangitis. All medical records of eligible patients were reviewed and the following parameters were collected: demographic data (age, gender), clinical data (aetiology of stent insertion), endoscopic parameters at the time of ERCP performance (incomplete stone extraction and complication of ERCP), period of follow-up, laboratory improvement after stent insertion) and follow up data on complications including cholangitis, stent related biliary pancreatitis, migration and biliary colic. Cholangitis was defined as new-onset right upper quadrant pain accompanied by fever and jaundice. Pancreatitis was defined as abdominal pain consistent with acute pancreatitis (acute onset of a persistent, severe, epigastric pain often radiating to the back) accompanied by elevated serum lipase or amylase activity at least three times greater than the upper normal limit and characteristic findings of acute pancreatitis on contrast-enhanced computed tomography or transabdominal ultrasonography [17]. Biliary colic was defined as new-onset transient colicky right upper quadrant pain accompanied by normal or abnormal liver enzymes and normal serum bilirubin level. All patients’ charts were extracted using ICD-9 codes for choledocholithiasis and cholangitis.

## 3. Study Endpoints

The primary endpoint was to compare the complications rate between all patients whose biliary stents were retained (patient refusal to remove or non-attendance to planned ERCP session for stent removal or replacement) for more than 6 months as being confirmed by imaging studies performed for assessment of stent presence, and patients whose stent was removed or replaced within 6 months after the index ERCP as recommended.

Secondary endpoints were to assess the complications rate at specific time intervals; for this purpose, we subdivided the study cohort into 2 comparison groups as follows: (0–12 months vs. >12 months and 0–18 months vs. >18 months). Furthermore, we aimed to assess the complications rate according to the type of the stent (plastic vs. metallic). The study was approved by the institution’s ethics committee (0034-16-SZMC obtained on 21.3.2016). Written informed consent was waived by the ethics committee due to the retrospective non-interventional nature of the study.

### 3.1. Types of Stents Used within Our Cohort

In our study we used two types of biliary stents, mostly plastic stents (Cotton-Huibregtse stents 10 Fr, 7 cm of Cook medical) and metallic stents (10-mm, 6 cm Wallflex of Boston Scientific). The inner tip of the stent was positioned in the CBD above the stone and its distal tip was positioned into the second part of the duodenum. Stent exchange was performed only when recurrent episode of cholangitis was experienced.

### 3.2. Statistical Analysis

Characteristics of participants are presented with descriptive statistics such as arithmetic means and standard deviation (SD) or range for continuous variables, or as frequencies (percentages) for categorical variables. The comparison of two independent groups was performed using Student’s *t*-test for continuous variables and the chi-square statistic for categorial variables. All tests applied were two-tailed. *p* value of 0.05 or less was considered to be statistically significant. Normality test was performed and showed normal distribution. All analyses were carried out using the statistical analysis software (SAS vs. 9.4 Copyright (c) 2016 by SAS Institute Inc., Cary, NC, USA).

## 4. Results

### 4.1. Baseline Demographics, Clinical and Laboratory Characteristics

**Figure 1:**
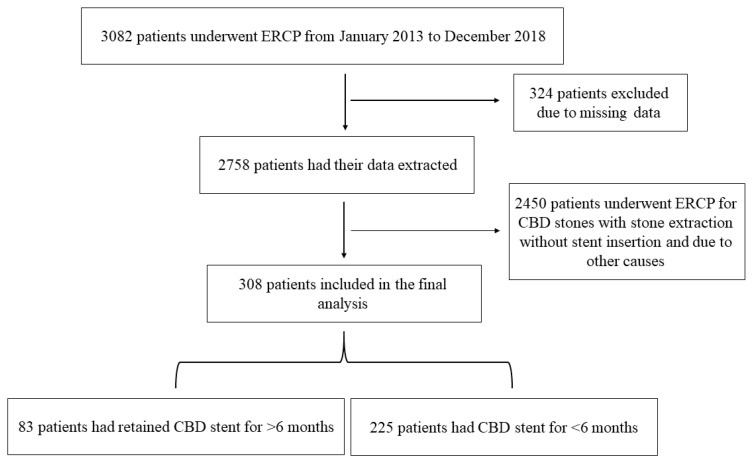
Demonstrating the flow chart of our study cohort.

A total of 3082 patients were identified from January 2013 to December 2018. Of these we included 308 patients with choledocholithiasis who had ERCP and biliary stent insertion. Eighty-three patients who underwent urgent ERCP for acute cholangitis complicating choledocholithiasis with biliary stent insertion mostly without papillotomy or with papillotomy when stone extraction was attempted, had retained long-term biliary stents of more than 6 months from insertion (group A) vs. 225 patients whose biliary stents were removed within 6 months following the index ERCP (group B), (Figure 1). The average ages of group A and B were 83.5 ± 4.8 years and 61.3 ± 17.9 years respectively (*p* < 0.001). Sixty-five percent and 63% of the patients were males in groups A and B respectively. None of the patients in group A had complete stone extraction, as 22.9% had incomplete stone extraction and 77.1% did not have their stone extracted. The causes of biliary stent insertion in group A were hemodynamic instability secondary to cholangitis (56 patients, 67.5%), technically difficult ERCP due to the presence of duodenal diverticula (25 patients, 30.1%) and inability to complete the ERCP due to sedation related complications (2 patients, 2.4%), while in group B, all patients had stent inserted as a preventive measure until performing cholecystectomy. The mean follow-up in group A was 66.1 ± 16.3 weeks (range 30–100 weeks) as compared to 11.1 ± 2.7 weeks (range 5–18) in group B. Clinical and endoscopic characteristics are demonstrated in Table 1.

### 4.2. Overall Complications during the Follow-Up Period

Table 2 and Table 3 demonstrate the rate of overall complications and each complication alone (cholangitis, stent related pancreatitis and biliary colic). The rate of overall complications during the follow-up period at the primary endpoint were similar between groups A and B (6% vs. 4.9%, OR 1.24, 95% CI 0.42–3.7, Chi square 0.69). Moreover, in the secondary endpoint analysis, comparing retained stents of <12 months vs. more than 12 months, again, there was no increase in the rate of all complication (5% vs. 6.1% respectively, OR 1.23, 95% CI 0.38–3.96, Chi square 0.72). Even after 18 months, the rate of all complications was similar to those who had retained stents of less than 18 months (5.3% vs. 5.2% respectively, OR 1.01, 95% CI 0.12–8.12, Chi square 0.98). Notably, the migration rate of biliary stent occurred in 5 patients (6%) in group A compared to 11 patients (4.9%) in group B (chi square 0.69). In spite of this, the complications rate was not higher in group A than in group B.

### 4.3. Sub-Analysis According to Each Complication Alone

There was no difference when we analyzed each complication alone. The rate of cholangitis, stent related pancreatitis and biliary colic were 3.6%, 1.2% and 1.2% vs. 2.7%, 0.44% and 1.8% in groups A and B respectively (Chi square 0.85). Moreover, in the secondary analysis, comparing retained stents of <12 months vs. more than 12 months, the rates of cholangitis, stent related pancreatitis and biliary colic were 2.8%, 0.4% and 1.65% vs. 3%, 1.5% and 1.5%, respectively (Chi square 0.8). Interestingly, the rate of each complication was not more common in patients with retained stents for more than 18 months (Chi square 0.4) (Table 2 and Table 3).

### 4.4. Complications Rate According to Stent Type

Two-hundred eighty-eight patients had plastic stents as compared to 20 patients who had metallic stents. Overall complications in the plastic stent group occurred in 15 patients (5.2%) as compared to 1 patient (5%) in the metallic group (OR 0.95, 95% CI 0.12–7.64, Chi square 0.97). Similarly, the rate of each complication alone was not different between the plastic stent vs. the metallic stent (chi square 0.07). Comparing patients according to stent type and stent retaining time, there was no difference between overall complications (OR 1.1, 95% CI 0.34–3.62, chi square 0.8) and each complication alone (chi square 0.86) in patients with plastic stents of more than 6 months as compared to less than 6 months. Similarly, patients with metallic stent of more than 6 months did not have either more overall complications (OR 1.1, 95% CI 0.92–1.29, chi square 0.4) or more of each single complication alone (chi square 0.56) as compared to patients with metallic stent of less than 6 months (Table 4 and Table 5).

## 5. Discussion

Our study demonstrated that approximately 10% of patients who underwent ERCP due to cholangitis complicating choledocholithiasis in the long-term (over 6 months in the primary endpoint) and even over 12 and 18 months in the secondary endpoints, retained biliary stents (plastic and metallic) and were not associated with more overall complications or any specific complication including cholangitis, pancreatitis and biliary colic compared to stent duration of less than 6 months. Actually, little is known about what happens when biliary stents are forgotten by patients for more than 6 months, despite several case reports of several dozens of patients reporting over 40% complications rate. The complications rates in our study were much lower (6% for overall complications and 3.6%, 1.2% and 1.2% for cholangitis, stent related pancreatitis and biliary colic, respectively) than previously reported in the literature.

A study by De Palma GD et al. of 49 patients with symptomatic choledocholithiasis and with irretrievable bile duct stone treated by endoscopic stenting for a median period of 39 months reported successful biliary drainage in all patients but found late complications in 40.8% of cases, with 3 cases of biliary-related death. They reached the conclusion that definitive biliary stenting for irretrievable stones should be relegated to highly selected cases [18]. Similarly, Bergman JJ et al. reported 40% complications rate following stent insertion for symptomatic choledocholithiasis, mostly cholangitis when biliary stents (polyethelene 10 Fr, 15 or 19 cm wedged in the intra-hepatic ducts) were inserted as permanent therapy in 58 patients for a median period of 36 months. They reached the same conclusion that permanent biliary stenting should preferably be restricted to patients unfit for elective treatment at a later stage and with a short life expectancy [16]. Similarly, Ang TL et al. reported the long-term outcome of plastic biliary stenting (7 Fr or 10 Fr) in 83 patients with symptomatic choledocholithiasis. In their report the complications rates for cholangitis, biliary colic, and pancreatitis were 71.4%, 3.6% and 3.6%, respectively. Notably, most of the stents used were 7 Fr in 79 patients, while only 4 patients had 10 Fr stents [19]. On the other hand, a recent retrospective study comparing the outcome in 3 groups of patients with choledocholithiasis unfit for definitive endoscopic stone removal or surgery using plastic biliary stenting (7 Fr, 7 cm), showed cholangitis rate of 2.9% and 8.6% in 6- and 12-month replacement groups, respectively, and 35.3% in the third group in whom stent replacement was carried out due to developing acute cholangitis with a median time for replacement of 16.3 months [20]. This study showed that the rate of acute cholangitis was low mainly in the 6 month replacement group, and increased as the stenting period increased, suggesting that 7 Fr plastic biliary stenting should be replaced every 6 months [20]. In comparison with the previous study, Slattery et al. showed a cholangitis rate in 7.4% with median stent patency of 11.8 months [21]. In our study, we showed a much lower complications rate even after a longer follow-up period. To the best of our knowledge, our study is the first to report these low complications rate probably due to the larger stent diameter and shorter length (10 Fr, 7 cm) that we used. In fact, most of the studies cited above [19,20,21] used 7 Fr stents, while one study reported the use of 10 Fr stent (15 or 19 cm wedged in the intra-hepatic ducts) [16]. In all studies the rates of complications were higher compared to our study, suggesting that both the stent diameter and length probably has a potential role in the development of stent related complications in the long-term. Notably, we did not find previous studies on the correlation between stent diameter and length with stent patency or complications, making our observation novel and necessitating further confirmatory studies.

Moreover, biliary stent migration rates have been reported to approach 4.9% and 5.9% for proximal (into the duct) and distal (out of the duct), respectively [22]. Similarly, the migration rate in our study was 6% in group A and 4.9% in group B. However, it is important to address that stent migration did not predispose for more stent related complications.

In our study, although more patients in group A had metallic stent compared to group B, after controlling for stent type, we found no difference in the rate of complications between the stents in the primary and the secondary endpoint analysis. Moreover, studies recommend to replace or remove a metallic stent after approximately 10 months from insertion [13]; in our study we found that stent duration of more than 12 months and even 18 months was not associated with more complications, again suggesting the long-term safety of metallic stenting. Interestingly, most of the patients in group A (94%) did not perform cholecystectomy in the follow-up period, though 37.3% of them had gallbladder stones without increase in the rate of overall complications, suggesting that biliary stenting is effective in preventing further episodes of cholangitis and even cholecystitis in the setting of gallbladder stones.

Biliary stenting for choledocholithiasis has been used mainly as a temporary measure to restore bile flow in cases of stone impaction in the CBD until stabilizing the patient’s condition followed by subsequent definitive endoscopic treatment or surgery [23]. With the rising age of our patient population, its associated comorbidities and prescribed medications including anticoagulants ant anti-aggregants, it has become nearly a daily dilemma which treatment suits the high-risk elderly comorbid patient. Demonstrating the safety of long-term biliary stenting (probably 10 Fr, 7 cm plastic stents or 10 mm, 6 cm metallic stents) in our study could provide an alternative safe and effective option for treating cholangitis complicating choledocholithiasis in this group of patients who are mostly elderly patients, adhering to one of the cardinal rules of practicing medicine “premium non nocere”.

This strategy also holds true for large, stiff, difficult-to-remove stones, where leaving a biliary stent seems to be an acceptable and safer option than insistence on removing an irretrievable stone, especially in high risk elderly comorbid patients [7,8,14].

Remarkably, our study results may have special implications in the current COVID-19 global pandemic period. Sadly, in response to COVID-19 pandemic most countries implemented lockdown as a major social distancing measure that naturally caused cancellation of elective gastrointestinal endoscopies as only emergent endoscopies were executed [24]. According to the recent position statement by the European Society of Gastrointestinal endoscopy (ESGE) regarding GI endoscopy units activity during COVID; only patients presenting with obstructive jaundice or ascending cholangitis should undergo urgent ERCP. Conversely, patients who are scheduled for elective biliary stent replacement were listed in the high priority procedure group (a procedure to be done immediately or postponed for 12 weeks [25]. Based on our results, postponing elective stent replacement is safe. Our study findings may provide ‘confidence’ to clinician and patients to postpone elective biliary stent during the lockdown period. However, prospective multicenter international studies are required to further assess this concern.

The main limitation of our study is its retrospective nature of data collection. On the other hand, to the best of our knowledge, this is the largest cohort of patients reported with retained biliary stents that were inserted for the indication of choledocholithiasis.

In conclusion: We found that long term biliary stenting for cholangitis complicating choledocholithiasis was effective, feasible and safe without increase in the complications rates during a long-term period. Factors associated with a low complication rate according to our study were larger stent diameter and probably shorter length. Therefore, we suggest a prolonged stenting policy for elderly patients or patients with comorbidities who are poor candidates for further definitive endoscopic or surgical treatments. Further multicenter, prospective studies with larger cohorts should be carried out to confirm our findings and better address this issue.

## Figures and Tables

**Table 1:** Demographics and endoscopic characteristics of study cohort.

Parameters	Group A (>6 Months)	Group B (<6 Months)
Number of patients	83	225
Age (years), Mean ±SD (range)	83.5 ± 4.8 (67–98)	61.3 ± 17.9 (20–93)
Gender, *N* (%)		
Male	54 (65.1)	142 (63.1)
Female	29 (34.9)	83 (36.9)
History of cholecystectomy, *N* (%)	23 (29.3)	0
Gallbladder stones, *N* (%)	31 (37.3)	164 (72.9)
Cause of stent insertion, *N* (%)		
Hemodynamic instability	56 (67.5)	0
Technically difficult ERCP	25 (30.1)	0
Sedation related complications	2 (2.4)	0
Preventive	0	225 (100)
Stone extraction during ERCP, *N* (%)		
No stone extraction	64 (77.1)	0
Incomplete stone clearance	19 (22.9)	0
Complete stone extraction	0	225 (100)
Complication of ERCP, *N* (%)		
None	72 (86.8)	209 (92.9)
Post ERCP pancreatitis	11 (13.2)	16 (7.1)
Bleeding	0	0
Perforation	0	0
Cholangitis	0	0
Mean follow-up in weeks, Mean ±SD (range)	66.1 ± 16.1 (30–100)	11.1 ± 2.7 (5–18)
Stent type, *N* (%)		
Plastic	71 (85.6)	217 (96.4)
Metallic	12 (14.4)	8 (3.6)
Normalization of liver enzymes during follow-up	65 (91.5)	160 (95.2)
Cholecystectomy performed during follow-up, *N* (%)	5 (6)	225 (100)

**Table 2:** Complications rate according to biliary stent duration.

	All Complications, N (%)	Cholangitis N, (%)	Stent Related Pancreatitis, N (%)	Biliary Colic, N (%)
Primary endpoint analysis				
<6 months	11 (4.9)	6 (2.7)	1 (0.44)	4 (1.8)
>6 months	5 (6)	3 (3.6)	1 (1.2)	1 (1.2)
Secondary endpoint analysis				
<12 months	12 (5)	7 (2.9)	1 (0.4)	4 (1.6)
>12 months	4 (6.1)	2 (3)	1 (1.5)	1 (1.5)
Secondary endpoint analysis				
<18 months	15 (5.2)	9 (3.1)	2 (0.7)	4 (1.4)
>18 months	1 (5.3)	0	0	1 (5.3)

**Table 3:** Chi square statistics of biliary stent related complications.

Endpoints	Chi Square Statistics
Primary endpoint analysis (<6 vs. >6 months)	
All complications	0.7
Each complication alone	0.8
Secondary endpoint analysis (<12 vs. >12 months)	
All complications	0.7
Each complication alone	0.8
Secondary endpoint analysis (<18 vs. >18 months)	
All complications	0.9
Each complication alone	0.5

**Table 4:** Complications rate according to biliary stent type.

Type of Stent	Plastic Stents		Metallic Stents	
	<6 vs. >6 Months		<6 vs. >6 Months	
Number of patients	217	71	8	12
All complications, *N* (%)	11 (5.1)	4 (5.6)	0	1 (8.3)
Cholangitis *N*, (%)	6 (2.7)	3 (4.2)	0	0
Stent related pancreatitis, *N* (%)	1 (0.46)	0	0	1 (8.3)
Biliary colic, *N* (%)	4 (1.8)	1 (1.4)	0	0

**Table 5:** Chi square statistics according to biliary stent type.

	Chi Square Statistics			
	All Complications		Each Complication Alone	
	Plastic	Metallic	Plastic	Metallic
Primary endpoint analysis < 6 vs. > 6 months	0.8	0.4	0.86	0.56

## Abbreviations

CBD	Common bile duct
CI	Confidence interval
ERCP	Endoscopic retrograde cholangiopancreatography
OR	Odds ratio
SD	Standard deviation

## References

[1] Canlas K.R., Branch M.S. (2007). Role of endoscopic retrograde cholangiopancreatography in acute pancreatitis. World J. Gastroenterol..

[2] Ahmed M., Kanotra R., Savani G.T., Kotadiya F., Patel N., Tareen S., Fasullo M.J., Kesavan M., Kahn A., Nalluri N. (2017). Utilization trends in inpatient endoscopic retrograde cholangiopancreatography (ERCP): A cross-sectional US experience. Endosc. Int. Open.

[3] Bor R., Madacsy L., Fabian A., Szepes A., Szepes Z. (2015). Endoscopic retrograde pancreatography: When should we do it?. World J. Gastrointest. Endosc..

[4] Lambert M.E., Betts C.D., Hill J., Faragher E.B., Martin D.F., Tweedle D.E. (1991). Endoscopic sphincterotomy: The whole truth. Br. J. Surg..

[5] Vaira D., D’Anna L., Ainley C., Dowsett J., Williams S., Baillie J., Cairns S., Croker J., Salmon P., Cotton P. (1989). Endoscopic sphincterotomy in 1000 consecutive patients. Lancet.

[6] Isayama H., Yasuda I., Tan D. (2017). Current strategies for endoscopic management of acute cholangitis. Dig. Endosc..

[7] Chan A.C., Ng E.K., Chung S.C., Lai C.W., Lau J.Y., Sung J.J., Leung J.W., Li A.K. (1998). Common bile duct stones become smaller after endoscopic biliary stenting. Endoscopy.

[8] Chopra K.B., Peters R.A., O’Toole P.A., Williams S.G., Gimson A.E., Lombard M.G., Westaby D. (1996). Randomised study of endoscopic biliary endoprosthesis versus duct clearance for bileduct stones in high-risk patients. Lancet.

[9] Bagul A., Pollard C., Dennison A.R. (2010). A review of problems following insertion of biliary stents illustrated by an unusual complication. Ann. R. Coll. Surg. Engl..

[10] Khashab M.A., Kim K., Hutfless S., Lennon A.M., Kalloo A.N., Singh V.K. (2012). Predictors of early stent occlusion among plastic biliary stents. Dig. Dis. Sci..

[11] Weickert U., Venzke T., Konig J., Janssen J., Remberger K., Greiner L. (2001). Why do bilioduodenal plastic stents become occluded? A clinical and pathological investigation on 100 consecutive patients. Endoscopy.

[12] Di Giorgio P., Manes G., Grimaldi E., Schettino M., D’Alessandro A., Di Giorgio A., Giannattasio F. (2013). Endoscopic plastic stenting for bile duct stones: Stent changing on demand or every 3 months. A prospective comparison study. Endoscopy.

[13] Nam H.S., Kang D.H. (2016). Current Status of Biliary Metal Stents. Clin. Endosc..

[14] Cotton P.B., Forbes A., Leung J.W., Dineen L. (1987). Endoscopic stenting for long-term treatment of large bile duct stones: 2- to 5-year follow-up. Gastrointest. Endosc..

[15] Sohn S.H., Park J.H., Kim K.H., Kim T.N. (2017). Complications and management of forgotten long-term biliary stents. World J. Gastroenterol..

[16] Bergman J.J., Rauws E.A., Tijssen J.G., Tytgat G.N., Huibregtse K. (1995). Biliary endoprostheses in elderly patients with endoscopically irretrievable common bile duct stones: Report on 117 patients. Gastrointest. Endosc..

[17] Banks P.A., Bollen T.L., Dervenis C., Gooszen H.G., Johnson C.D., Sarr M.G., Tsiotos G.G., Vege S.S. (2013). Classification of acute pancreatitis—2012: Revision of the Atlanta classification and definitions by international consensus. Gut.

[18] De Palma G.D., Galloro G., Siciliano S., Catanzano C. (2001). Endoscopic stenting for definitive treatment of irretrievable common bile duct calculi. A long-term follow-up study of 49 patients. Hepatogastroenterology.

[19] Ang T.L., Fock K.M., Teo E.K., Chua T.S., Tan J. (2006). An audit of the outcome of long-term biliary stenting in the treatment of common bile duct stones in a general hospital. J. Gastroenterol..

[20] Tohda G., Dochin M. (2018). Management of endoscopic biliary stenting for choledocholithiasis: Evaluation of stent-exchange intervals. World J. Gastrointest. Endosc..

[21] Slattery E., Kale V., Anwar W., Courtney G., Aftab A.R. (2013). Role of long-term biliary stenting in choledocholithiasis. Dig. Endosc..

[22] Johanson J.F., Schmalz M.J., Geenen J.E. (1992). Incidence and risk factors for biliary and pancreatic stent migration. Gastrointest. Endosc..

[23] Sherman S., Hawes R.H., Lehman G.A. (1990). Management of bile duct stones. Semin. Liver Dis..

[24] Repici A., Pace F., Gabbiadini R., Colombo M., Hassan C., Dinelli M., Maselli R., Spadaccini M., Mutignani M., Gabbrielli A. (2020). Endoscopy Units and the Coronavirus Disease 2019 Outbreak: A Multicenter Experience from Italy. Gastroenterology.

[25] Gralnek I., Hassan C., Beilenhoff U., Antonelli G., Ebigbo A., Pellise M., Arvanitakis M., Bhandari P., Bisschops R., van Hooft J. (2020). ESGE and ESGENA Position Statement on gastrointestinal endoscopy and COVID-19: An update on guidance during the post-lockdown phase and selected results from a membership survey. Endoscopy.

